# Atropia in Acute Coryza

**Published:** 1886-02

**Authors:** 


### Atropia in Acute Coryza.

Dr. R. Gray, in the *Medical News*, December 5, 1885, says the first case in which he used atropia in acute coryza, was that of a man in middle life, who had "caught" a severe "cold in his head" several days previously. When he came for advice the disease had reached an extreme stage. There were severe frontal headache, a hot, burning sensation in the nose, forehead and cheeks, there was some conjunctivitis, and very profuse muco-purulent discharge, which was extremely irritating. The skin about the nose was irritated and inflamed, and the general condition was one of great misery. Atropia was given with the idea of decreasing the amount of the discharge. The dose was $\frac{1}{120}$ of a grain, repeated after four hours. It had a most marked effect, and the next day the patient was quite free from headache, heat, and swelling, and from discharge.

Since then the remedy has been tried in a large number of cases, in all stages of the disease, and at all ages, with uniform success. It is now his established practice, and is preferable to cocaine in this, that no local application is needed to the nose, thus saving a very painful manipulation.

The only objection that has been made to the treatment, is where the eyesight is troubled. But the dose needed to cure the coryza is not sufficient to produce much disturbance of vision. It is only necessary to influence the secretion, and an extreme degree of dryness of the throat and nasal passages is of no advantage.

